# Ten simple rules for successfully managing EU research grants

**DOI:** 10.1371/journal.pcbi.1012335

**Published:** 2024-08-15

**Authors:** David G. Pina

**Affiliations:** European Research Executive Agency, European Commission, Brussels, Belgium; Carnegie Mellon University, UNITED STATES OF AMERICA

## Introduction

There have been several Ten Simple Rules (TSR) articles addressing the preparation and drafting of research grant proposals, giving general advice to potential applicants [1] and more specifically providing tips for writing grants in emerging countries [2] or for preparing postdoctoral fellowships’ applications [3,4]. However, so far, no TSR article has focused on the challenges that one may face in the post-grant award phase. This article aims at filling that gap, by listing key points to take into consideration when managing and implementing research grants, from the pre-grant signature phase up to the closure of the project. Therefore, it should be of particular interest for any (new) project coordinators, grant managers, research administrators, and other staff involved in grant support services.

Considering that each research funding agency has a set of different regulations and practices in relation to the preparation, awarding, implementation, and monitoring of grants, I decided to focus this TSR article on the specific case of research grants awarded under the European Union’s current framework programme for research and innovation, Horizon Europe (HE) [5,6]. This funding instrument has a massive budget of 95 billion Euros, covering the year 2021 to 2027 [7]. By the end of its third year of implementation (2021 to 2023), more than 10,000 EU research grants had been signed (average of 2.9 million Euro per grant), involving more than 20,000 participating institutions [8]. Among HE subprogrammes, the European Research Council (ERC) and the Marie Skłodowska*-*Curie Actions (MSCA) are among the most competitive and well established, attracting many applicants from all scientific domains, in a so-called bottom-up approach [9]. In contrast, grants awarded under the 6 clusters of HE Pillar 2 (addressing Global Challenges and European Industrial Competitiveness) fund specific topics in predefined research domains, such as health, culture, climate change, energy, food, bioeconomy, natural resources, and environment, among others [10]. More than 80% of the EU research grants consist of international consortia of multiple organisations/institutions—also called beneficiaries—which are mostly universities, research centres, and private companies [8]. HE funds are available not only for institutions from EU countries, but also for institutions located in the so-called associated countries, which include neighbouring countries (e.g., Norway, Iceland, Ukraine, Turkey, Israel) and also the United Kingdom (since 2024), Canada, New Zealand, and soon South Korea. Moreover, funding is available for participating institutions from low-income countries of Africa, Asia, and Latin America. Therefore, this article can be of great help to researchers and managers from non-EU countries, newly involved in EU research grants.

The ERC Starting, Consolidator and Advanced grants, and the MSCA postdoctoral fellowships, awarded respectively to individual Principal Investigators and postdoctoral researchers, are simpler to manage from an administrative point of view as they do not entail the coordination and communication challenges inherent to large international consortia. However, most of the points listed below can apply to them too.

Rules 1 to 3 apply to the whole grant management lifecycle. Rules 4 to 6 cover the aspects related to the preparation of the Grant Agreement (GA), which involves the gathering and collection of all the necessary technical, legal, financial, and administrative information and documentation leading to the GA signature. Finally, Rules 7 to 10 address the post GA signature phase, i.e., the implementation of the project properly speaking, and its coordination and management over its lifespan.

## Rule 1: Establish excellent relations with your assigned EU project officer or manager

Congratulations, your proposal has been selected for funding! Soon after learning about this positive outcome, the funding agency will invite you to prepare the GA. You will be provided with details of the EU project officer (PO) who will accompany you in this process. The PO will be your main contact point for all matters related to the GA preparation and in most cases for the subsequent implementation of your project. Over the lifespan of your project, your PO can change. Make sure to establish and maintain with them the best possible professional and working relationship, as you will have to consult them on many occasions for any relevant grant-related issue, especially those needing prior approval.

Remember that the PO will want your project to be a success as much as you do, so consider them as an ally willing to help find solutions to possible obstacles or difficulties appearing over the lifecycle of the project. From the beginning, take the time for a proper introductory phone or video call together (usually the PO will take this initiative) and to agree on the way you will interact with each other. You should define the frequency of your contacts, what type of issues need their attention, etc. You should see the PO as a facilitator and catalyst whose goal is to ensure you will implement the project successfully, delivering the expected outcomes and impact, and respecting the rules and conditions defined in the GA. All in all, you should see the PO almost as an (external) member of your team!

## Rule 2: Foster an effective, collaborative environment between your partners and be ready to handle possible conflicts

Engage all your team members and people who will be involved in your project from the start. You should put in place a series of best practices allowing the relevant information to flow naturally between all of you. Plan and have regular meetings with your partners to discuss the work plan, tasks, activities, and possible issues. The periodicity and arrangements of such meetings should be agreed beforehand by the team. With the development of a plethora of collaborative IT tools such as Google Workspace or Microsoft Teams, international research teams working on common projects can now benefit from improved communication channels.

Over the lifetime of your project, it is likely that some conflict will appear at some stage, be it for scientific reasons or disagreements, a collaborator dropping out of the project, or for some observed misconduct or misbehaviour. If issues arise among some of your partners which may put at risk the implementation and successful achievement of specific project tasks, you should act as a mediator and facilitator, trying to find compromises and accommodate possible solutions, such as the redistribution of tasks and responsibilities among the other members of the team. If necessary, it may be useful to have a neutral stakeholder (external to the project) intervene. The devil is in the details, and you may find out too late, for instance, that not all the partners are using the same parametrisation, methodologies, protocols, endpoints, and other important determinants of the experimentation. Synergy demands that all results of the consortium are interoperable and consistent so that they can contribute to a reliable conclusion or product.

Conflict resolution is a key skill that project managers should acquire [11]. It is important to bear in mind that conflict is not always negative, as it can bring new ideas or solutions to problems, if well managed and handled at the project level. Remember that ultimately, it is the whole consortium that is responsible for the proper implementation of the project. If some of the partners do not play according to the rules of the game, you may need to seek replacements (via an amendment to the GA—see **Rule 6**).

## Rule 3: Surround yourself with the right people

Most probably, you will not be able to be on top of all issues regarding your project. This is why you should surround yourself by people with the appropriate training, background, and/or professional experience. This will allow you to delegate some tasks you may feel less comfortable with. Enrol people with diverse profiles, as it takes more than just scientists to properly manage a research grant. Depending on the size and complexity of your project, you may wish to have a dedicated project manager, who would deputise and take the lead in all daily nonscientific aspects of the grant. You also need people with budgetary, financial, and legal background, and administrative support is often necessary.

You can find these profiles within your organisation’s horizontal services, or if not available, you could alternatively develop these project management competences and skills within your own team members if they show interest. Entrusting some of your team members with specific, nonscientific tasks could turn out to be very handy and diversify their career prospects [12–13]. The European Association of Research Managers and Administrators (EARMA) offers training courses and organises regular events and conferences for professionals involved in grant management. Other certifications on project management, partially suitable for managing research grants, are provided by specialised organisations, such as the Project Management Institute (PMI) and the Project Management Methodology (PM^2^) developed by the European Commission (with a series of freely accessible tools and publications).

Outside your team or institution, a group of so-called National Contact Points (NCPs) is appointed at national level to provide personalised support at both pre- and post-grant award stages. Therefore, they can be a good source of support and information in all matters related to EU research grants. Your project may address issues related to innovation, access rights, copyright, data protection, licensing, patenting, etc. In case your institution does not have a dedicated Innovation or Technology Transfer Office (TTO) to support you with these specialised topics, you can seek advice from the European IP Helpdesk.

## Rule 4: Get familiar with the resources and tools available

Good news for you: All the relevant information and tools that you need to properly manage your grant is usually provided on the funding agency’s website. In general terms, you will find the necessary documentation, such as guidelines and templates covering the whole grant implementation lifecycle, namely the awarding phase and GA preparation, project monitoring and reporting, payments, and grant closure.

For the specific case of EU research projects, there is a one-stop shop website: the “EU Funding & Tenders” Portal (F&T Portal), which acts as a single entry point for all applicants and grantees of EU projects. This website is where you will find answers to all your questions, besides those you will discuss with the PO (**Rule 1**). At this point in time, you should already be familiar with this website, as it is the same one used by applicants to submit their proposals. However, when you get awarded an EU grant, you will start consulting and reading other parts of the F&T Portal. In the “Guidance & documents” section you will have access to the “Reference documents” where all the key HE documents can be found, such as the applicable legislation, Work Programmes, and also the model GA, which refers not only to the so-called general model GA, but also the model GA for Unit Costs (applicable for MSCA) and Lump Sum, which are simplified type of grants. This section also includes a series of Guidance documents and Templates & forms, namely reporting templates.

The Online Manual (OM) is the interactive step-by-step online guide where you will find all the practical information you need to know in relation to the project implementation workflows. A complementary tool to the Online Manual is the IT How To, especially its section entitled “Manage your grant,” which contains a series of hyperlinks showing a multitude of walkthroughs and screenshots of the different steps to follow. The F&T Portal also includes a long list of Frequently Asked Questions (FAQ) with a series of filters allowing you to check for common and recurrent questions. You can use keywords to help you in the search and apply relevant filters (e.g., Grant preparation and signature, etc.), depending on what type of answers you are looking for. In case you still need further help or if you face some IT issues when working in the F&T Portal, you can always contact the IT Helpdesk, via the Helpdesk & Support section. It is worth knowing that there is multitude of useful videos and presentations available on a dedicated YouTube channel for EU Science & Innovation, many of which address the post-award grant management business, such as those entitled Horizon Implementation Day and HE Coordinators’ Day. A list of such events is kept up to date in the F&T Portal’s event page. Finally, it is worth mentioning that most probably, depending on the experience that your institution has in dealing with research grants, you may have at your disposal a dedicated intranet webpage or some institutional supporting material at hand. So do not hesitate to ask around for support.

## Rule 5: Engage in the preparation of the Grant Agreement and familiarize yourself with its nonscientific content

The tasks related to the GA preparation will be the first set of actions you will have to coordinate and perform. Although it may seem like purely administrative work for a researcher, it is essential that you get properly involved in this process as it will help you to better understand what it entails to manage (for the first time) an EU research grant. In principle, you will be given few weeks to provide relevant technical, financial, legal, and administrative information prior to the GA signature.

For the specific case of EU research grants, the OM dedicated page on Grant Preparation is a good starting point for getting acquainted with this process. As the whole workflow is done electronically (paperless), do not hesitate to consult the respective IT How To page, which contains explanations on all the necessary steps, and includes screenshots of what needs to be done in the F&T Portal. After a few (possible) iterations with the PO, you will agree on a final version of the Description of Action (DoA). This document will be an annex to the GA and mostly consists of the scientific and technical content of the project, and what should be performed and achieved by whom and when. In an EU research grant, the DoA is, in general terms, the transposition of the initial research proposal, divided in 2 parts. The Part A includes the list of participants (i.e., beneficiaries), work packages, staff efforts (quantified in terms of person-months per beneficiary and per work package), deliverables and milestones (and their respective due dates), critical risks (see **Rule 6**), and reviews (see **Rule 7**). The Part B includes the remaining elements of the initial proposal, structured into 3 sections: Excellence, Impact, and Implementation, plus an additional section at the end addressing the ethics and security issues of the project, if relevant. Remember that the original content of the proposal should remain intact, as changes at GA preparation stage are only foreseen in exceptional circumstances, when clarifications or missing details are needed (and requested by the PO). In most cases, the EU services will aim at signing the GA within 3 to 4 months. During this period, if not yet done before, you will need to confirm that all beneficiaries have been validated (via the Participant Register), meaning that beneficiaries have been registered properly as legal entities. They will have to appoint their representatives (via their Legal Entity Appointed Representative—LEAR) and need to understand the rules related to these processes. Make sure they react in time in case you need contributions from them. All the partners (GA beneficiaries) will have to submit a Declaration of Honour (DoH) and will have to accede to the GA after its signature (via Accession forms). The process of GA signature is well explained in the respective OM and IT How To dedicated pages. In parallel to this process, you will have to prepare a Consortium Agreement (**Rule 6**). All in all, this exercise will be an opportunity for you to surround yourself with the right people (**Rule 3**) and to properly kick-off the project. While getting involved in the GA preparation, you will soon realise that managing EU research grants is not only about conducting research work and delivering scientific results. It also means keeping an eye on the bigger picture, achieving the expected impact, communicating to the public, or in some cases, providing training to researchers. When signing a GA, the consortium of beneficiaries and the funding agency formalise a contractual relationship. Consequently, the beneficiaries accept and agree to implement the action under their own responsibility, abiding by the GA’s clauses, obligations, terms, and conditions. Therefore, it is crucial to take some time to read and get minimally acquainted with the different GA sections, chapters, articles, and annexes. For example, you should pay particular attention on the type (form) of grant that you have been awarded (specified under GA Article 5), as different financial rules apply depending on whether your project is funded in the form of a so-called mixed actual (real) costs grant, unit grant, or lump sum grant. Your key reference document for understanding and interpreting everything related to the GA is the Annotated Grant Agreement (AGA).

If you managed previous grants under Horizon 2020, the predecessor of HE that was implemented between 2014 and 2020, you should take the time to check the differences in the GA structure and content. As a researcher, you do not need to know all of it in detail, but you should at least grasp and understand how it is structured. Additionally, it is essential to realise that the GA contractual obligations are institutional, as it is the beneficiaries, i.e., organisations, that are signatories (parties) to the GA, and not individual persons. This is why the GA is not signed by the scientific leader or manager of the project directly, but by a legal representative of the institution, e.g., dean, vice dean, director, or any person with an equivalent status and capacity to legally represent the organisation.

The current model GA for EU grants includes a new section, named “Data Sheet,” summarising some of the key features and elements applicable to the grant in question. It is important that not only the coordinator, but also all the partners (other beneficiaries) of the consortium, understand which costs are eligible and which are not, what requires an amendment to the GA, and what does not, what are the rules for carrying out the activities foreseen in the DoA, what type of documents and information should be kept for possible checks, reviews, or audits, etc. (**Rule 8**). Remember that the research project, which was transposed into the DoA, is just one part of the whole GA. It is an essential part of it, containing the tasks and activities to be performed during the project implementation, however, ignoring the rest of the GA that covers the legal, financial, and administrative rules applicable for the whole lifecycle of the project could cause great harm. Everything you will do in relation to the DoA will have to be compliant with the rest of the GA. It would be a mistake for grant recipients to play down their legal obligations and to not be aware of the “rules of the game.” For example, reading (at least once) the provisions of the GA chapter 5 will make you aware of all the actions foreseen in case of noncompliance with the GA obligations. Once again, surrounding yourself with competent people having relevant (in this case legal) background will help (**Rule 3**). Do not hesitate to get in touch with the legal, financial, and administrative support services of your institution to receive additional guidance or internal instructions.

## Rule 6: Prepare a solid Consortium Agreement, mitigate risks, and be aware of what can be changed in the Grant Agreement

For projects with several participants (multi-beneficiary), you will need to prepare and sign a Consortium Agreement (CA), because the GA covers the terms and conditions (rights and obligations) between the parties only, meaning the EU funding agency on one side, and all the beneficiaries and other participants, on the other side. It does not regulate the terms and conditions among the beneficiaries (partners) themselves. This is where the CA steps in, as a complement to the GA, by defining how beneficiaries will work together, outside of their common obligations towards the funding agency. The CA must be signed by all your institutional partners that addresses organisational issues, decision-making, distribution of roles and responsibilities, and how to solve internal issues and difficulties you may encounter during the implementation of the project. It also usually tackles financial aspects of the grant and, when relevant, issues related to intellectual property or ownership between partners. This is especially relevant knowing that some complex grants may involve dozens of beneficiaries from a multitude of countries. The funding agency is not a party to the CA and will not sign it. Because of that, you will have to verify that any provision included in the CA will not contradict any of the GA provisions, as in case of conflicting provisions, the latter would prevail and take precedence over the CA. There are useful templates available for preparing a CA, the DESCA model being the most commonly used for EU research grants (and other adaptations of that model to specific type of grants, such as for the MSCA or Lump sum grants).

To find effective solutions for possible obstacles or problems that may appear over the course of the project, pay particular attention to the critical risks that you identified in the original proposal application, and the mitigation measures you have put in place to address them. Do not wait until the last minute to communicate difficult issues to the PO. You should consult them in case you deviate from the initial plan (as described in the DoA), to seek advice on how to proceed with the project. Changes to the content of the DoA are possible, but they should remain reasonable, well justified, and acceptable, and not put into question the initial award decision. There is an Amendment Guide available in the F&T Portal reference documents, which lists the type of amendments that are possible to implement. Always discuss these options with the PO before initiating any change (**Rule 1**) and seek advice from your institution grants office or legal support service.

## Rule 7: Make the most of the kick-off meeting and project reviews

In most cases, the first milestone after the start of your project will be the kick-off meeting. It is crucial that you prepare it carefully and properly. It will allow all the partners to meet each other. It may bring a larger audience of relevant stakeholders as well, such as the PO and other actors who will be involved in your project, in one way or another, such as support staff from the institutions involved, policy officers, and/or legal representatives. Depending on the scope and size of your grant, the kick-off meeting may involve also other interest groups at local, regional, or national level and may be open, at least partially, to the public in general.

Take maximum advantage of this meeting and ensure that you cover all aspects related to grant implementation and management. Agree about the agenda with the PO and leave enough time for questions and answers from the partners. In this meeting, you will have the opportunity to discuss with the rest of the partners not only the scientific and research goals of the project, but also the aspects related to the management of the grant. Have a diversified set of speakers, covering also the legal, financial, and administrative context of what it entails to be part of an EU research grant. A common mistake of some grant recipients is to tend to focus only on the scientific goals of the project, and to disregard other relevant aspects from the GA obligations. Do not forget to present the roles and responsibilities of each partner, such as Work Packages (WP) and task leaders, and the timeline of the milestones and deliverables to be achieved. The Consortium Agreement should also be presented (**Rule 6**).

In most cases, over the grant lifespan, one or more project review(s) will be held. In these reviews, the research team members and other people involved in the project will discuss the progress of the work performed. Usually, the PO is supported by external experts, in their task of monitoring the implementation of the project. These experts are selected based on their scientific knowledge and expertise in the field. Most often, these reviews will be connected to the contractual reporting exercises, defined in the GA (see **Rule 10**). They occur according to an agreed timeline and consist of an assessment of the proper implementation of the project in line with the DoA and compliance with the GA obligations. Once again, consider these meetings as an opportunity to check how well the project is progressing, to identify best practices and successes, to discuss possible issues, and to take corrective measures, if necessary. The experts will produce a project review report addressed to the consortium, to which you will be able to react and comment.

## Rule 8: Keep track of your project’s activities and be prepared for audits

As soon as your project starts, it is important to put in place some IT tools, platforms, repositories, or structured folders in which you will keep records of the activities developed and implemented throughout the lifetime of your grant. It is of utmost importance to be able, at any point in time, to provide proof or evidence of the work performed, in case it is requested by the funding agency or audit services.

In the specific case of EU research grants, the tasks and activities to be implemented during the lifetime of your project are detailed in the DoA and structured into different WP. Each WP has a number of planned deliverables—some of which will become public domain, while some others will remain confidential, depending on their level of sensitivity—to be submitted as scheduled in the DoA of the GA. These deliverables usually are the result of WP-related specific tasks. In case your DoA does not include a Gantt chart with the timeline of the activities to be implemented throughout the lifetime of the project, it is advisable to create one (or a similar tool) that will allow you to monitor the activities scheduled over time. Make sure your dedicated repositories or project folders are safe and secured, to store there all your relevant documents and use an adequate IT tool to keep a structured overview of the project’s activities. Beneficiaries have the contractual obligation to keep records and supporting documents usually up to 5 years after the end of the project in case they need to document proper implementation (GA Article 20). Do not forget that these records and documents may also be needed for checks and reviews (**Rule 7**) and can be requested for audits too (GA Article 25). Ensure that all the partners participate and commit to keep track of their tasks. It is common that partners tend to concentrate on scientific tasks and overlook other activities, such as those related to communication, dissemination, and exploitation (**Rule 9**). The coordinator must be able to monitor and report on all the activities of the project, collect outputs, and request contributions from all beneficiaries involved in a particular WP.

## Rule 9: Communicate, disseminate, and exploit the results of your project

There are several TSR articles addressing science communication [14,15], innovative dissemination practices [16], online outreach [17], and results exploitation [18], stressing the relevance of such activities in the research endeavour. Hence, in order to achieve the highest scientific, societal, economic, and environmental impact, it is essential to communicate about your project, to promote it, and to disseminate and exploit any results or outputs deriving from it.

In the context of EU research grants, the communication, dissemination, and exploitation activities are contractual obligations (see GA article 17 and Annex 5). The EU funding must be properly acknowledged, and any communication or dissemination related to your project should include a disclaimer. The European Commission has developed several tools to support beneficiaries in their communication, dissemination, and exploitation activities [19–21], namely the Horizon Results Booster and the Innovation Radar. Depending on its size, your institution most probably has some specialised services dealing with these topics, such as an institutional communication office, a press or public relations office, a TTO, a public research data repository, and/or (online) library. All of which can be of great help in supporting the management of your project.

The HE Programme Guide, which is a reference document for the preparation of proposals, has dedicated sections addressing the dissemination and exploitation of research results and Open Science. Research data management should be in line with the FAIR principles [22], and open access to research outputs is a contractual requirement. As such, a Data Management Plan (DMP) is expected as a deliverable, usually due at the beginning of the project (and regularly updated afterwards). The EU services provide detailed information and a template on how to prepare such document in the F&T Portal, under the project reporting templates and in the HE Programme Guide. The importance of a proper research data management has been acknowledged [23], and a previous TSR article gives advice on how to create a good DMP [24].

## Rule 10: Give sufficient attention to grant reporting obligations

The reporting exercises are key milestones falling under the project management responsibilities, as they inform the funding agency and the PO about the progress made in a given period, to assess the quality of the work performed, to acknowledge possible delays or deviations from the original plan, and to check and validate the costs you are claiming. Each funding agency will have specific requirements concerning its grants reporting features, all of which are contractually defined.

For the specific case of EU research grants, the submission of periodic reports comes on top of the continuous reporting obligations (see GA Article 21), which consists mostly of the submission of deliverables, milestones, and research outputs on a regular and timely manner, according to the schedule defined in the DoA. The OM has a dedicated section on the continuous reporting tasks.

Should you not have the time (or discipline) to deal with the continuous reporting yourself, consider hiring a project or grant manager, full- or part-time. If this is not possible, ask for administrative support from your organisation (**Rule 3**). But be aware that a skilled or experienced project manager will certainly ease and relieve your workload. They will not replace you in your role of project coordinator but will guarantee effective and smooth continuous reporting.

Be aware that it is only upon the approval of the periodic reports that the amounts you received as prefinancing will be cleared and further intermediate and final payments can follow. The submission of periodic reports is done electronically via your personal page of the F&T Portal, under Grants -> My project(s). Periodic reports are due according to the schedule defined in the GA Data Sheet. Do not underestimate that a significant part of the reporting exercise in a multi-beneficiary consortium consists of collecting information and contributions from each of the partners, so anticipate and plan these tasks well ahead of time and use the existing templates available in the F&T Portal to prepare the reports.

## Conclusions

When managing research grants, it is not enough to perform good science, you need to ensure that what you do is compliant with the conditions defined by the funding agency and that you follow the “rules of the game,” including those related to communication, dissemination, and exploitation of research results. By implementing an EU research grant, you are using European taxpayers’ money. Hence, it is logical that you keep overall control and supervise the management of the grant, and remain responsible and accountable towards the funding agency. A good use of resources is key, in order not only to produce research outputs, but also deliver on the broader outcomes and ultimately deliver the societal impacts expected in the Work Programmes and call topics.

In multi-beneficiary consortia, the project scientific coordinator or leader does not need to be the same person as the administrative and financial coordinator. Legally speaking, the responsibilities linked to the coordination of EU research projects refer to activities of an administrative, financial, and technical nature. This means that the rules listed above may be addressed to different individuals within the same project, with very different backgrounds and levels of understanding of grant management. This is why it is crucial to define some principles to which all stakeholders involved can sign up to and make certain that they possess good leadership skills [25]. Hopefully, this article will raise awareness and help coordinators and other researchers or managers of EU research grants to fully understand what it entails to manage these types of grants.

## References

[1] Bourne PE, Chalupa LM. Ten Simple Rules for Getting Grants. PLoS Comput Biol. 2006;2(2):e12. doi: 10.1371/journal.pcbi.0020012 16501664 PMC1378105

[2] de Oliveira DM, Buckeridge MS, dos Santos WD. Ten Simple Rules for Developing a Successful Research Proposal in Brazil. PLoS Comput Biol. 2017;13(2):e1005289. doi: 10.1371/journal.pcbi.1005289 28151931 PMC5289455

[3] Yuan K, Cai L, Ngok SP, Ma L, Botham CM. Ten Simple Rules for Writing a Postdoctoral Fellowship. PLoS Comput Biol. 2016;12(7):e1004934. doi: 10.1371/journal.pcbi.1004934 27415752 PMC4945040

[4] Baumert P, Cenni F, Antonkine ML. Ten simple rules for a successful EU Marie Skłodowska-Curie Actions Postdoctoral (MSCA) fellowship application. PLoS Comput Biol. 2022;18(8):e1010371. doi: 10.1371/journal.pcbi.1010371 35980892 PMC9387847

[5] Regulation (EU) 2021/695 of the European Parliament and of the Council of 28 April 2021 establishing Horizon Europe–the Framework Programme for Research and Innovation, laying down its rules for participation and dissemination. OJ L170/1.

[6] Horizon Europe, the EU research and innovation programme 2021–27. 2021. Publications Office of the European Union, Luxembourg. ISBN 978-92-76-29646-1. doi: 10.2777/601756 Available from: https://op.europa.eu/s/y6ia.

[7] Horizon Europe, Budget. 2021. Publications Office of the European Union, Luxembourg. ISBN 978-92-76-31601-5. doi: 10.2777/202859 Available from: https://op.europa.eu/s/y6h9.

[8] Horizon Europe implementation–Key Figures for 2021–2023. 2023. Publications Office of the European Union, Luxembourg. ISBN 978-92-68-16156-2. doi: 10.2777/646835 Available from: https://op.europa.eu/s/zK2C.

[9] Horizon Europe, pillar I–Excellent science. 2021. Publications Office of the European Union, Luxembourg. ISBN 978-92-76-24767-8. doi: 10.2777/456952 Available from: https://op.europa.eu/s/y6ig.

[10] Horizon Europe, pillar II—Global challenges and European industrial competitiveness. 2021. Publications Office of the European Union, Luxembourg. ISBN 978-92-76-24768-5. doi: 10.2777/881197 Available from: https://op.europa.eu/s/y6ib.

[11] Lewitter F, Bourne PE, Attwood TK. Ten Simple Rules for avoiding and resolving conflicts with your colleagues. PLoS Comput Biol. 2019;15(1):e1006708. doi: 10.1371/journal.pcbi.1006708 30657752 PMC6338354

[12] Vlasits AL, Smith ML, Maldonado M, Brixius-Anderko S. Supporting nonlinear careers to diversify science. PLoS Biol. 2023;21(9):e3002291. doi: 10.1371/journal.pbio.3002291 37708100 PMC10501578

[13] Zatz M, Dupere S. Careers in Science and Grant Administration: View from the National Institutes of Health. Cold Spring Harb Perspect Biol. 2018;10(9)a032847. doi: 10.1101/cshperspect.a032847 30181194 PMC6120704

[14] Borowiec BG. Ten simple rules for scientists engaging in science communication. PLoS Comput Biol. 2023;19(7):e1011251. doi: 10.1371/journal.pcbi.1011251 37471282 PMC10358936

[15] Bautista C, Alfuraiji N, Drangowska-Way A, Gangwani K, de Flamingh A, Bourne PE. Ten simple rules for improving communication among scientists. PLoS Comput Biol. 2022;18(6):e1010130. doi: 10.1371/journal.pcbi.1010130 35737640 PMC9223317

[16] Ross-Hellauer T, Tennant JP, Banelytė V, Gorogh E, Luzi D, Kraker P, et al. Ten simple rules for innovative dissemination of research. PLoS Comput Biol. 2020;16(4):e1007704. doi: 10.1371/journal.pcbi.1007704 32298255 PMC7161944

[17] Bik HM, Dove ADM, Goldstein MC, Helm RR, MacPherson R, Martini K, et al. Ten Simple Rules for Effective Online Outreach. PLoS Comput Biol. 2015;11(4):e1003906. doi: 10.1371/journal.pcbi.1003906 25879439 PMC4399988

[18] Fletcher AC, Bourne PE. Ten Simple Rules To Commercialize Scientific Research. PLoS Comput Biol. 2012;8(9):e1002712. doi: 10.1371/journal.pcbi.1002712 23028299 PMC3459878

[19] European Research Executive Agency. 2023. Communication, Dissemination & Exploitation. Publications Office of the European Union, Luxembourg. ISBN: 978-92-95080-88-1. doi: 10.2848/289075 Available from: https://op.europa.eu/s/y6kq.

[20] European IP Helpdesk 2019. Making the most of your H2020 project. Publications Office of the European Union. ISBN: 978-92-9202-494-9. doi: 10.2826/045684 Available from: https://op.europa.eu/s/zK3U.

[21] European IP Helpdesk. 2022. Successful valorisation of knowledge and research results in Horizon Europe. Publications Office of the European Union. ISBN: 978-92-9469-287-0. doi: 10.2826/437645 Available from: https://op.europa.eu/s/y6kp.

[22] Wilkinson MD Dumontier M, Aalbersberg IJ, Appleton G, Axton M, Baak A, et al. The FAIR Guiding Principles for scientific data management and stewardship. Sci Data. 2016;3:160018. doi: 10.1038/sdata.2016.18 26978244 PMC4792175

[23] Kanza S, Knight NJ. Behind every great research project is great data management. BMC Res Notes. 2022;15(1):20. doi: 10.1186/s13104-022-05908-5 35063017 PMC8781028

[24] Michener WK. Ten Simple Rules for Creating a Good Data Management Plan. PLoS Comput Biol. 2015;11(10):e1004525. doi: 10.1371/journal.pcbi.1004525 26492633 PMC4619636

[25] Bourne PE. Ten simple rules for good leadership. PLoS Comput Biol. 2022;18(6):e1010133. doi: 10.1371/journal.pcbi.1010133 35679224 PMC9182262

